# Venetoclax combined with ATRA shows promising therapeutic potential for TFG:: RARA variant APL: a case report

**DOI:** 10.3389/fonc.2025.1529640

**Published:** 2025-05-02

**Authors:** Xianfeng Ouyang, Jianguo Yan, Su Hu, Wenfeng Zhu, Qibao Zhou, Fei Hu

**Affiliations:** ^1^ Department of Hematology, Jiujiang First People’s Hospital, Jiujiang, Jiangxi, China; ^2^ Department of Cardiovascular Medicine, Jiujiang First People’s Hospital, Jiujiang, Jiangxi, China

**Keywords:** acute promyelocytic leukemia, variant APL, TFG::RARA, venetoclax, all-trans retinoic acid, arsenic trioxide

## Abstract

Most cases of acute promyelocytic leukemia (APL) are driven by the PML::RARA fusion gene, which is sensitive to differentiation induction therapy comprising of all-trans retinoic acid (ATRA) and arsenic trioxide (ATO). Treatment with ATRA plus ATO has achieved remarkable clinical outcome in patients with typical APL. However, 5% of patients still died from relapsed/refractory disease, predominantly high-risk APL and variant APL. The diagnosis and treatment of variant APL remain challenging. Here, we report a case of TFG::RARA variant APL recognized by targeted RNA sequencing. The patient achieved a sustained complete response following treatment with venetoclax combined with ATRA. Currently, the overall survival (OS) of the patient has exceeded 30 months, and the progression-free survival (PFS) has reached 29 months. Our results suggest that venetoclax combined with ATRA may be an ideal treatment option for patients with TFG::RARA variant APL.

## Background

Acute promyelocytic leukemia (APL) is a subtype of acute myeloid leukemia (AML) with unique morphological and cytogenetic features. It is classified as AML-M3 in the French-American-British (FAB) classification system and accounts for 10–15% of newly diagnosed AML (1). Classic APL is characterized by a specific chromosomal balanced reciprocal translocation t(15;17) (q24.1;q21.2) that generates the oncogenic fusion protein PML::RARA, resulting in abnormally proliferation of immature promyelocytes in the bone marrow (2–6). The past 30 years have seen rapid progress in classical APL treatment by using all-trans retinoic acid (ATRA) and arsenic trioxide (ATO) to target the PML::RARA fusion protein, converting the disease from highly lethal to highly curable (7, 8).

However, approximately 2% of patients are variant APL, characterized by atypical rearrangements, including two scenarios: RARA is fused to other partners instead of PML and PML::RARA fusion protein is negative, or the translocation refers to other RAR family members rather than RARA (8, 9). RARB rearrangement and RARG rearrangement were also found to cause APL (2, 9). In addition to PML, RARA has many other partner genes that interact to produce fusion proteins, such as PLZF::RARA, NPM1::RARA, NUMA::RARA, STAT5B::RARA, and BCOR::RARA (10–17). Apart from high-risk APL, primary variants of APL that are mostly resistant to ATRA and ATO (9). The identification, diagnosis, and treatment of patients with APL variants present considerable challenges for clinicians.

Herein, we report a patient with TFG::RARA variant APL treated with venetoclax plus ATRA to achieve a favorable curative effect. To the best of our knowledge, this is the second report of such a variant APL till submission. However, we employed a distinct therapeutic schedule and performed a longer follow-up period.

## Case presentation

**Figure 1:**
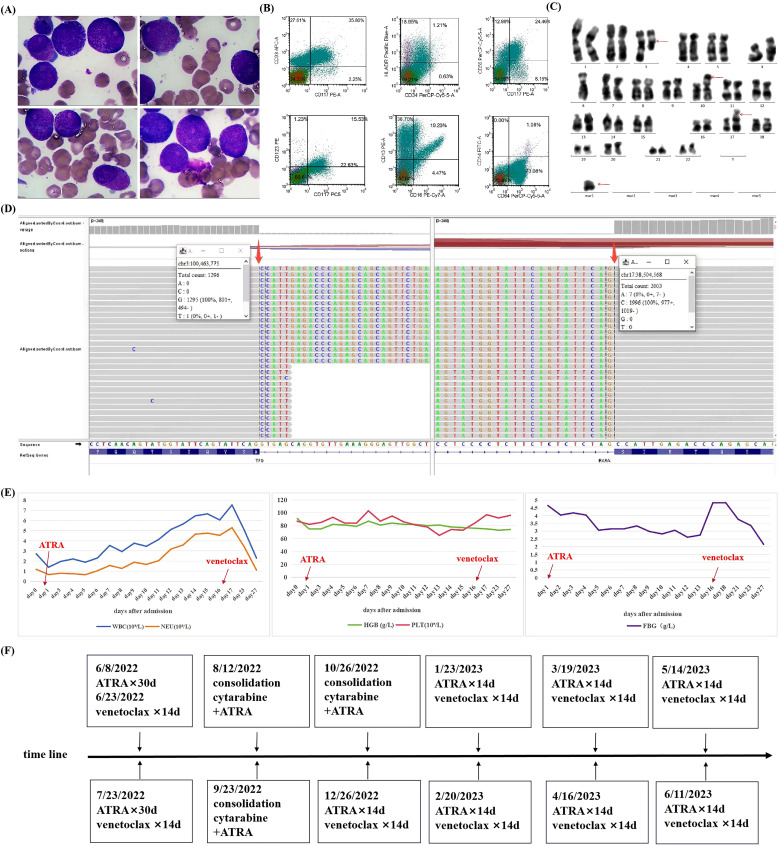
Clinical characteristics of a patient with variant acute promyelocytic leukemia with TFG::RARA rearrangement **(A)** Wright–Giemsa staining of bone marrow smear (×1000). **(B)** Immunophenotype of blasts/promyelocyte by flow cytometry. **(C)** Chromosome G band analysis, with red arrows indicating abnormal karyotypes. **(D)** Targeted RNA sequencing of TFG::RARA fusion gene, with positive fusion genes identified by thick red arrows. Targeted RNA sequencing for blood tumors involves isolation of RNA, conversion into cDNA, enrichment of tumor-specific genes, and sequencing to identify mutations and gene expression changes. **(E)** Changes in blood cells and fibrinogen during the first course of induction therapy. **(F)** Timeline with the main treatment of the clinical episode. WBC, white blood cell; NEU, neutrophil; HGB, hemoglobin; PLT, platelet; FBG, fibrinogen; ATRA, all-trans retinoic acid.

A 56-year-old female patient presenting with general pain for 3 months visited the outpatient clinic. Blood routine examination showed a white blood cell (WBC) count of 2.71×10^9^/L (ref. 3.5–9.5×10^9^/L) without abnormal immature cells, neutrophil ratio of 43.9%(ref. 40%-75%), hemoglobin level of 91g/L (ref.115–150 g/L), and platelet count of 87×10^9^/L (ref.125-350×10^9^/L). And then the patient was admitted to the hematology department because of pancytopenia. The blood clotting function displayed a fibrinogen level of 4.65 g/L (ref. 2.00–4.00 g/L), D-dimer level of 5.76 ug/mL (ref. 0.00–0.55 ug/mL), along with prothrombin time and activated partial thromboplastin time were 13.0 seconds (ref. 10.0–14.0 s) and 26.6 s (ref. 24.0–34.0 s), respectively. Lactate dehydrogenase (LDH) was 334 U/L (ref. 135–225U/L) and alpha-hydroxybutyrate dehydrogenase (HBDH) was 278U/L (ref. 72–182U/L). There were no abnormalities in liver and kidney function. Bone marrow smear showed 50.6% abnormal hypergranular promyelocytes without Auer rods (**Figure 1A**). Immunophenotypes of the blasts/promyelocytes by flow cytometric were as follows: CD34-, CD117+, CD33+, CD13+, HLA-DR (weak) +, CD64+, CD56+, CD9+, CD123(dim) +, CD14-, CD2-, CD5-, CD7-, CD19-, CD20- (**Figure 1B**). An initial diagnosis of APL was established, and 25 mg/m^2^ ATRA was administered on day 1 of admission.

The PML::RARA rearrangement was negative upon quantitative polymerase chain reaction (q-PCR) and fluorescence *in situ* hybridization (FISH). Chromosome analysis revealed a 46, XX, t (3;17) (q21; q25), -6, add (10) (p13), +mar (6)/46, XX (12) karyotype (**Figure 1C**). Next-generation sequencing identified Shwachman-Bodian-Diamond syndrome (SBDS) gene mutations, although clinical relevance was not established. Thereafter, the specimen was subjected to targeted RNA sequencing, a technique focusing on a specific RNA molecule or a set of genes. The sequencing results revealed the presence of the TFG::RARA fusion gene with a breakpoint in exon 7 of the TFG gene and exon 3 of the RARA gene (**Figure 1D**). The patient was eventually diagnosed with a variant APL. Owing to the lack of therapeutic target for ATO, induction therapy for the TFG::RARA variant APL comprised venetoclax and ATRA. The specific protocols used were ATRA 20 mg twice daily (30 days), venetoclax 100 mg once daily (14 days), and voriconazole 200 mg q12h to enhance the blood concentration of venetoclax. During induction therapy, the patient did not develop notable differentiation syndrome or bleeding, nor warrant transfusions of blood products. Specific changes in blood cell counts and fibrinogen levels are shown in **Figure 1E**. Follow-up of bone marrow morphology and measurable residual disease (MRD) by flow cytometric after one course of induction therapy indicated complete remission (CR). Subsequently, the same regimen was repeated for another course of induction therapy. In addition to bone marrow morphology and MRD, targeted RNA sequencing was negative for the TFG::RARA rearrangement, and the chromosomal karyotype returned to normal. After two courses of ATRA and venetoclax therapy, the patient achieved genetic remission.

Consolidation therapy comprised three courses of cytarabine (1.5 g/m^2^ every 12 hours, on day 1, day 3, and day 5, respectively) combined with ATRA (20 mg twice daily for 30 days). Maintenance regimen included ATRA 20 mg twice daily and venetoclax 100 mg once daily for 14 days, with voriconazole 200 mg administered every 12 hours to increase the blood concentration of venetoclax (18). The treatment process of the patient is illustrated in **Figure 1F**. The patient remained in continuous CR at follow-up, as determined by bone marrow morphology, MRD, targeted RNA sequencing and chromosome karyotype analysis. The last reexamination was conducted on 7 November 2024. The results of the bone marrow morphology, MRD, and TFG::RARA fusion gene were negative, with normal blood routine, coagulation function, and the chromosome karyotype. Currently, the patient has an overall survival (OS) exceeding 30 months.

## Discussion

In addition to high-risk APL, the existence of variant APL has been identified as an underlying factor for APL relapse and refractoriness. If bone marrow morphology and immunotypes reveal a typical APL phenotype but a negative PML::RARA fusion gene, variant APL needs to be identified through fusion gene screening and/or targeted RNA-sequencing as early as possible. In the current case, the patient who presented with pancytopenia without bleeding, differing from the clinical manifestations of typical APL. A missed diagnosis was feasible owing to the normal coagulation function and absence of abnormal cells in the peripheral blood smear. Bone marrow smears showed classic abnormal hypergranular promyelocytes, while flow cytometry revealed a typical APL immunophenotype. However, neither q-PCR nor FISH detected the PML::RARA rearrangement. Accordingly, targeted RNA sequencing of hematological tumors is particularly important. Therefore, the patient was eventually diagnosed with TFG::RARA variant APL.

TFG::RARA was first reported by Chong et al. in 2018 (19). The protein encoded by the *TFG* gene plays a role in maintaining the dynamic state of the endoplasmic reticulum and its associated microtubules (20, 21). Likewise, TFG can also form fusion genes together with other partner genes, such as TFG::TEC, TFG::RET, TFG::NTRK1, and TFG::ALK (22–25). The first case of TFG::RARA variant APL involved a 16-year-old male who presented with leukopenia, moderate anemia, normal platelet count, and normal fibrinogen levels. We hypothesized that the TFG::RARA variant APL exerts a relatively limited effect on coagulation function and results in less severe thrombocytopenia. Therefore, these two patients with TFG::RARA variant APL did not present notable bleeding symptoms or diffuse intravascular coagulation (DIC). Nevertheless, this requires validation using large-sample data. Treatment of variant APL remains challenging. Chong et al. (19) employed an ATRA single-drug induction therapy and verified *in vitro* that TFG::RARA is sensitive to ATRA *in vitro*. Subsequently, the authors administered two courses of ATRA combined with idarubicin followed by maintenance with ATRA alone. At the time of submission, their patient was still undergoing maintenance therapy; hence, the long-term survival of the patient remains unclear.

To date, only one case of TFG::RARA variant APL has been reported, and its overall prognosis remains poorly understood. Notably, our case belonged to the CD56-positive APL subtype associated with a poor prognosis (26), and treatment warranted careful consideration. Currently, experience in treating variant APL patients is insufficient, and chemotherapy with or without ATRA has been used for treatment in the past (9). Venetoclax, a type of selective B‐cell lymphoma 2 (BCL‐2) inhibitor, has been widely used in AML patients (27). Furthermore, venetoclax‐based treatment was reportedly effective in patients with ATRA/ATO‐resistant APL (28, 29). Additionally, some case reports revealed that venetoclax can achieve good results in ATRA/ATO-resistant variant APL patients (30–34). Xu et al. (35) reported that venetoclax can overcome resistance to ATRA in the case of TNRC18::RARA variant APL. BCL-2 functions in cooperation with PML-RARA fusion protein to promote acute leukemia and prevent neutrophil differentiation (36). BCL-2 may be associated with ATRA resistance in APL patients (37). Therefore, we performed induction therapy with two courses of venetoclax plus ATRA. The patient achieved CR after the first course of induction therapy. Moreover, TFG::RARA and chromosome tests were negative after two- courses induction therapy. Given the uncertain prognosis of this variant APL, we administered three courses of consolidation chemotherapy comprising cytarabine combined with ATRA referring to high-risk APL. We then performed maintenance therapy with venetoclax combined with ATRA for 7 months. The patient has been off treatment for more than one year and has maintained CR status. Currently, the OS is more than 30 months and the progression-free survival (PFS) has reached 29 months. Our case revealed that venetoclax combined with ATRA to treat of TFG::RARA variant APL could achieve excellent curative effects.

## Conclusion

In this case report, we diagnosed and treated a patient with TFG::RARA variant APL, the second case reported till submission. Our results demonstrate that venetoclax combined with ATRA can be a good therapeutic option for patients with TFG::RARA variant APL. Nevertheless, data on the efficacy of ATRA plus venetoclax compared with that of ATRA alone are lacking to confirm the additional benefits of venetoclax on disease control and relapse.

## Data Availability

The raw data supporting the conclusions of this article will be made available by the authors, without undue reservation.
